# Single monosomy as a relatively better survival factor in acute myeloid leukemia patients with monosomal karyotype

**DOI:** 10.1038/bcj.2015.84

**Published:** 2015-10-16

**Authors:** J E Jang, Y H Min, J Yoon, I Kim, J-H Lee, C W Jung, H-J Shin, W S Lee, J H Lee, D-S Hong, H-J Kim, H-J Kim, S Park, K-H Lee, J H Jang, J S Chung, S M Lee, J Park, S K Park, J-S Ahn, W-S Min, J-W Cheong

**Affiliations:** 1 Division of Hematology, Department of Internal Medicine, Severance Hospital, Yonsei University College of Medicine, Seoul, South Korea; 2 Department of Hematology, Cancer Research Institute, Seoul St Mary's Hospital, College of Medicine, The Catholic University of Korea, Seoul, South Korea; 3 Division of Hematology-Medical Oncology, Department of Internal Medicine, Cancer Research Institute, Seoul National University College of Medicine, Seoul, South Korea; 4 Department of Hematology, Asan Medical Center, University of Ulsan College of Medicine, Seoul, South Korea; 5 Division of Hematology/Oncology, Department of Medicine, Samsung Medical Center, Sungkyunkwan University School of Medicine, Seoul, South Korea; 6 Division of Hematology-Oncology, Department of Internal Medicine, School of Medicine, Medical Research Institute, Pusan National University Hospital, Busan, South Korea; 7 Department of Hemato-Oncology, Department of Internal Medicine, Inje University Busan Paik Hospital, Busan, South Korea; 8 Department of Internal Medicine, Gachon University Gil Medical Center, Gachon University School of Medicine, Incheon, South Korea; 9 Department of Internal Medicine, Division of Hematology & Oncology, Soonchunhyang University Bucheon Hospital, Bucheon, South Korea; 10 Department of Hematology and Oncology, Chonnam National University Hwasun Hospital, Hwasun, South Korea

## Abstract

Monosomal karyotype (MK) defined by either ⩾2 autosomal monosomies or single monosomy with at least one additional structural chromosomal abnormality is associated with a dismal prognosis in patients with acute myeloid leukemia (AML). It was detected in 174 of 3041 AML patients in South Korean Registry. A total of 119 patients who had received induction therapy were finally analyzed to evaluate the predictive factors for a positive prognosis. On multivariate analysis, single monosomy, the absence of abn(17p), ⩾10% of cells with normal metaphase and the achievement of a complete remission (CR) after induction therapy were significant factors for more favorable outcomes. Especially, single monosomy remained as a significantly independent prognostic factor for superior survival in both patients who received allogeneic hematopoietic stem cell transplantation (allo-HSCT) in CR and who did not. Allo-HSCT in CR improved overall survival significantly only in patients with a single monosomy. Our results suggest that MK-AML may be biologically different according to the karyotypic subtype and that allo-HSCT in CR should be strongly recommended to patients with a single monosomy. For other patients, more prudent treatment strategies should be examined. Furthermore, the biological mechanism by which a single monosomy influences survival should be investigated.

## Introduction

Although several different cytogenetic classifications exist for acute myeloid leukemia (AML), it has been generally agreed that specific cytogenetic abnormalities result in unfavorable prognoses. Adverse cytogenetic risk factors include −5/5q deletion (del(5q)), −7/7q deletion (del(7q)), −17/17p abnormality (abn(17p)), inv(3)(q21q26/t(3;3)(q21;q26) and complex karyotype (CK).^1, 2, 3, 4^ Recently, monosomal karyotype (MK) has been shown to be associated with a dismal prognosis in AML, and it has gotten another prognostic value in AML patients compared with CK.^5, 6, 7, 8, 9^ This new category is defined by either the presence of two autosomal monosomies or one monosomy with at least one additional structural chromosomal abnormality (in the absence of core-binding factor AML and acute promyelocytic leukemia).^5^ Although a higher percent of cells with normal metaphases or absence of abn(17p) or −5/del(5q) in MK-AML may be associated with prognosis,^10, 11^ studies for clinical significance according to the karyotypic heterogeneity or subtype of MK is limited. The benefits of allogeneic hematopoietic stem cell transplantation (allo-HSCT) in patients with MK have also been controversial. Several retrospective analyses have suggested that allo-HSCT would be associated with improved survival.^12, 13^ In contrast, Kayser *et al.*^7^ reported no significant benefit from allo-HSCT in patients achieving complete remission (CR) after induction therapy.

Therefore, to clarify the predictors of improved outcome and to determine appropriate indication for allo-HSCT for MK-AML patients, this study investigated the influence of specific clinical and karyotypic characteristics on prognosis, as well as proper therapeutic strategies for MK-AML patients.

## Patients and methods

### Patients

For this study, nationwide database of Korean AML Registry, which has been operated from 2007 by the Korean Society of Hematology AML/Myelodysplastic Syndrome Working Party, was analyzed. A total of 3041 AML patients from 28 institutions were registered at the time of analysis. The cohort included 1679 male and 1356 female, with a median age of 51 years (range, 16–87 years). AML was diagnosed according to the World Health Organization definition of >20% blasts in the bone marrow (BM) or peripheral blood.^14^ Patients without cytogenetic analysis, those in whom cytogenetic analysis failed and those with core-binding factor abnormalities or acute promyelocytic leukemia were excluded from this study. From January 2007 to December 2011, 174 patients (5.7%) who met the definition of MK-AML were selected for this study, and finally 119 patients from 10 institutions who received induction therapy were retrospectively analyzed. The study protocol was approved by each institution's institutional review board.

### Cytogenetic and molecular analysis

Cytogenetic analysis was performed using metaphasic cells from BM aspirates obtained at diagnosis using the conventional G-banding method. Karyotype designation was based on the International System for Human Cytogenetic Nomenclature.^15^ Only clonal abnormalities were considered positive results. Abnormalities were considered clonal if ⩾2 metaphases had the same aberration in the case of a structural abnormality or an extra chromosome, or if ⩾3 metaphases shared the same abnormality in the case of a monosomy. CK was defined as ⩾3 clonal abnormalities or ⩾4 clonal abnormalities. The MK was defined as the presence of two autosomal monosomies or one monosomy with at least one additional structural chromosomal abnormality, as previously reported by Breems *et al.*^5^

### Statistical analysis

The distribution of patients' characteristics between groups was compared using the *χ*^2^ or Fisher's exact tests for categorical variables and the Mann–Whitney *U*-test for continuous variables. Overall survival (OS) was defined as the time from the date of AML diagnosis to the date of death or the last follow-up. Event-free survival (EFS) was defined for all patients and was measured from the date of AML diagnosis until treatment failure, relapse from CR or death from any cause, whichever occurred first. Relapse-free survival (RFS) in patients achieving CR after induction chemotherapy was calculated from the date of CR achievement until the date of relapse or death from any cause. When comparing the survival of patients who underwent allo-HSCT, OS and EFS were calculated from the date of allo-HSCT. Logistic regression was used to test for the factors associated with the achievement of CR in univariate and multivariate analyses. A Kaplan–Meier survival analysis was performed to estimate the probabilities of survival. Differences in survival between groups were compared using the log-rank test. Factors affecting OS, EFS and RFS were analyzed using the Cox proportional hazards model in univariate and multivariate analyses. *P<*0.05 was defined as statistically significant. All statistical analyses were performed using SPSS, version 20.0 (SPSS Inc., Chicago, IL, USA).

## Results

### Patient characteristics

The baseline characteristics of 119 MK-AML patients are summarized in Table 1. The median age was 56 years (range, 17–82 years); 83 patients (69.7%) were male and 36 patients (30.3%) were female. Nineteen patients (16%) were secondary AML developed following exposure to cytotoxic agents or as a subsequent event in another hematologic disorder, and most patients (89.1%) had CK (⩾3 clonal abnormalities). The most frequent cytogenetic abnormalities were −7/7q deletion (47.1%), and −5/5q deletion (41.2%) and 17p abnormality (17.6%) were followed. MK defined by one single autosomal monosomy with at least one structural chromosomal abnormality was detected in 44 patients (37%, single monosomy group), and MK defined by ⩾2 autosomal monosomies was detected in 75 patients (63%, ⩾2 monosomy group). Monosomies could be detected in every chromosome in the ⩾2 monosomy group, but chromosomes 1, 3, 4, 6, 10, 15, 19, 21 and 22 were not affected in the single monosomy group. Patients in the ⩾2 monosomy group were significantly older and exhibited lower white blood cell and platelet counts compared with those in the single monosomy group (Table 1). Most patients in the ⩾2 monosomy group exhibited CK with a higher incidence of abn(17p) and −5/del(5q) compared with those in the single monosomy group. In contrast, the incidence of inv(3)/t(3;3) tended to be higher in the single monosomy group. There were no other significant differences in the clinical characteristics between two groups.

### Therapeutic strategies and patient response

**Figure 1:**
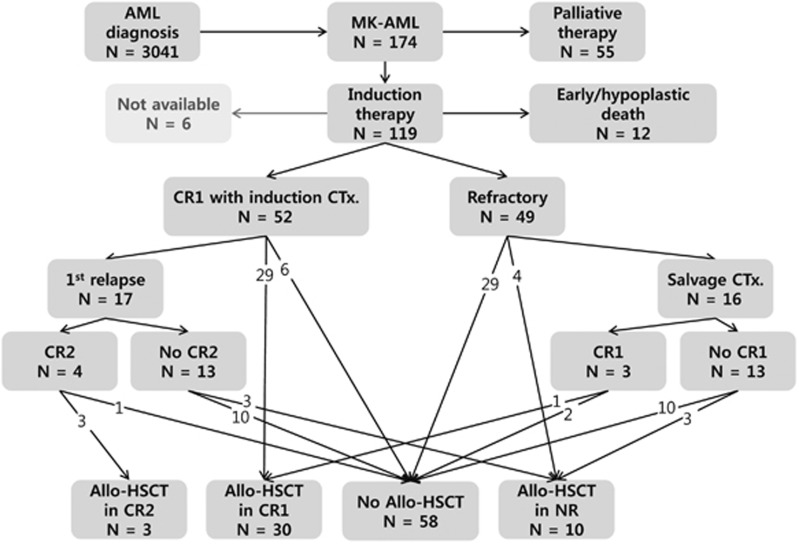
Flow diagram of patients. CTx, chemotherapy; NR, non-remission.

Patients received either one or two courses of myelosuppressive induction chemotherapy; 108 (90.8%) received daunorubicin or idarubicin in combination with cytarabine or the cytarabine analog, *N*4-behenoyl-1-β-d-arabinofuranosylcytosine, 6 (5%) received cytarabine combined with etoposide and 5 (4.2%) received other chemotherapy regimens. Except 6 patients who were not available for assessment, 52 (46%) attained CR in response to induction therapy (Figure 1). As a postremission treatment, patients received 1–6 cycles of consolidation chemotherapy according to each institution's policy. Early/hypoplastic death occurred in 12 patients (10.6%) and 49 (43.4%) exhibited a refractory response to induction therapy. Finally, 43 patients underwent allo-HSCT: 33 of whom achieved CR status (32 patients achieved CR1 or CR2 status after successful induction therapy and 1 patient achieved CR1 with salvage chemotherapy after failing two cycles of induction therapy), whereas the remaining 10 patients had either relapsed or demonstrated a refractory response at the time of allo-HSCT. The median time interval between diagnosis and allo-HSCT was 4.7 months (range 2.4–13.3 months). The type of donor was an HLA-matched sibling in 13 patients (30.2%), an HLA-matched unrelated donor in 20 (46.5%) and a haploidentical donor in 10 (23.3%). As a conditioning regimen, myeloablative regimens were used for 20 patients (46.5%), and reduced intensity conditioning regimens based on fludarabine was for the rest. Granulocyte-colony-stimulating factor mobilized peripheral blood stem cells in the majority of stem cell source (86.0%).

### Prognostic factors for the response to induction therapy

Supplementary Table 1 illustrates the response of patients to induction therapy and the affecting factors. CR rate decreased with age as a numerical variable (1-year old, *P*=0.008), and the presence of ⩾10% of cells with normal metaphase was another good prognostic factor for CR after induction therapy (*P=*0.002) in univariate analysis. Older age (⩾60 years) and secondary AML were associated with lower CR rate of 37% and 26.3%, respectively, although this was not significant. The number of monosomies did not impact patients' response to induction therapy (*P=*0.758). In multivariate analysis, a younger age (*P=*0.023) and the presence of ⩾10% of cells with normal metaphase still significantly correlated with a higher rate of CR achievement (*P=*0.005). The factors associated with a higher incidence of early/hypoplastic death included the percent of cells with normal metaphase (*P=*0.033) and the presence of abn(17p) (*P=*0.037) (Supplementary Table 2).

### Prognostic factors for survival outcome

**Figure 2:**
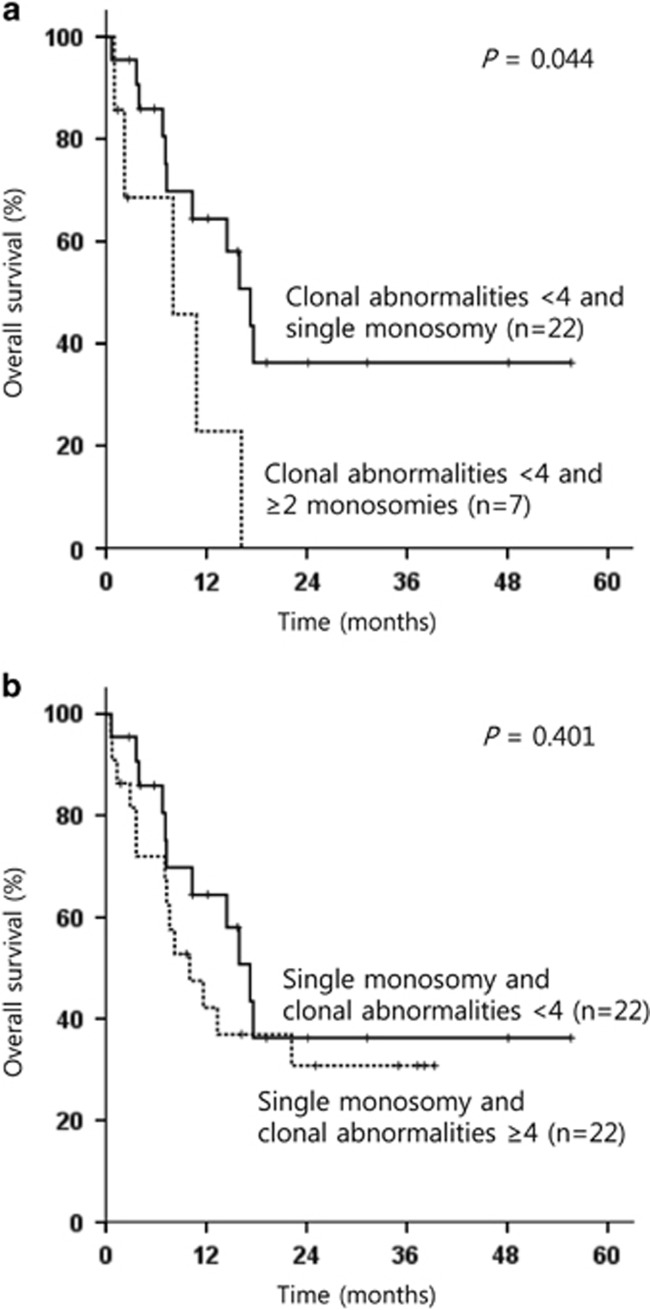
OS of (**a**) patients not having complex karyotype (⩾4) according to monosomal karyotype subtype, and (**b**) patients with single monosomy according the degree of clonal abnormalities (⩾4 versus <4).

The median follow-up time was 39.4 months from diagnosis. The median OS and EFS were 8.1 months (95% confidence interval (CI), range 6.5–9.8 months) and 4.6 months (95% CI, range 3.1–6.1 months), respectively (Supplementary Table 3). The 3-year OS and EFS rates were 19.6% and 7.3%, respectively. Interestingly, previously well-known prognostic factors for AML, including a high white blood cell count at diagnosis, subtype of AML, CK and adverse cytogenetic abnormalities, with the exception of abn(17p), did not show any influence on OS (Supplementary Table 3). Age <60 years, the achievement of CR after induction therapy, single monosomy subtype, the presence of ⩾10% of cells with normal metaphase and the absence of abn(17p) were associated with better OS in univariate analysis. CK (⩾4 clonal abnormalities) tended to affect survival outcome, although this was not statistically significant. In multivariate analysis, the achievement of CR after induction therapy, single monosomy subtype, the presence of ⩾10% of cells with normal metaphase and the absence of abn(17p) remained independent prognostic factors for better OS (Table 2). The achievement of CR after induction therapy (*P*<0.001), single monosomy (*P*=0.019) and the diagnosis of *de novo* AML (*P*=0.027) significantly correlated with higher EFS rates in multivariate analysis. Next, the positive impact of single monosomy subtype in patients without CK (⩾4 clonal abnormalities) was analyzed, and single monosomy subtype has kept its positive prognostic impact on OS in patients without CK (*P*=0.044; Figure 2a). Conversely, in the single monosomy group, CK did not impact OS (*P*=0.401; Figure 2b).

### The beneficial effect of allo-HSCT in MK-AML

**Figure 3:**
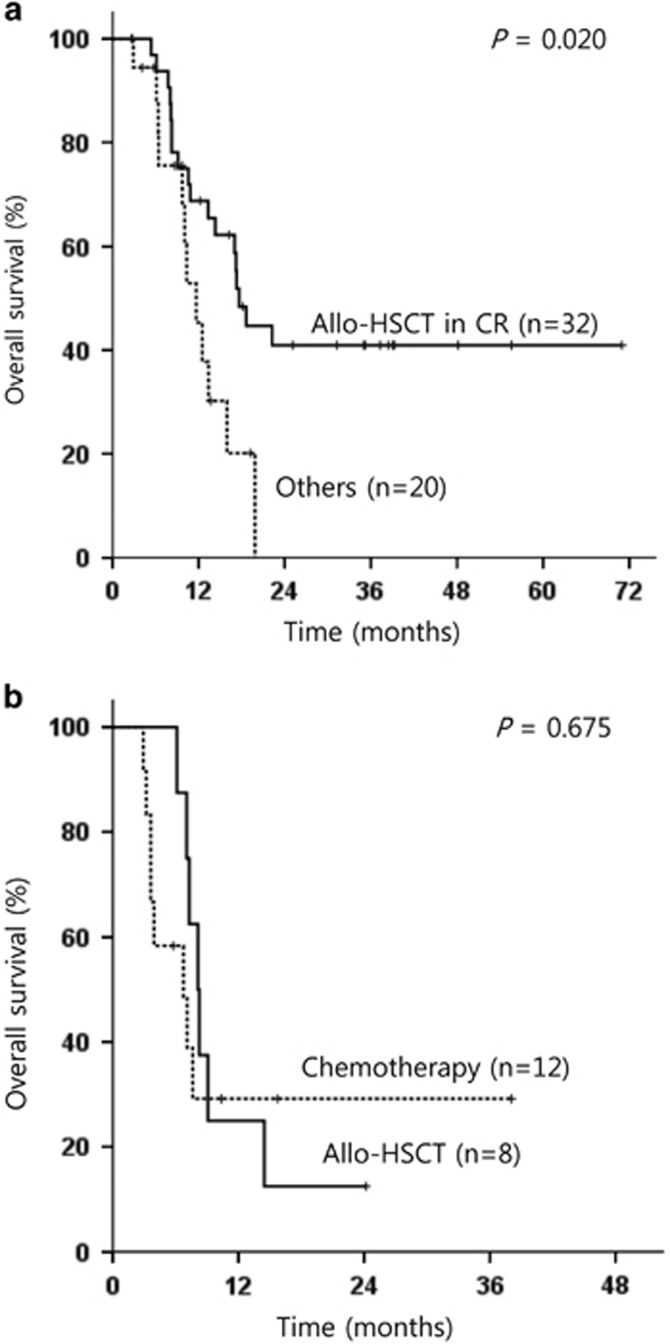
Kaplan–Meier curves for OS (**a**) according to the receipt of allo-HSCT for consolidation in patients who achieved CR after induction therapy and (**b**) according to the receipt of allo-HSCT as salvage treatment in patients who did not achieve CR after induction therapy.

Of 52 patients achieving CR after induction therapy, 32 underwent allo-HSCT in CR; 13 (59%) of 22 patients with single monosomy and 19 (63%) of 30 patients with ⩾2 monosomies underwent allo-HSCT in CR. Table 3 shows the result of multivariate analyses for OS and RFS in patients who achieved CR after induction therapy. In multivariate analysis, single monosomy subtype (hazard ratio (HR): 0.314, 95% CI: 0.135–0.732; *P*=0.007) and allo-HSCT in CR (HR: 0.268; 95% CI: 0.090–0.798; *P*=0.018) were independent predictive factors for better OS (Table 3). Although allo-HSCT in CR improved survival in patients achieving CR after induction therapy (*P=*0.020; Figure 3a), allo-HSCT as a salvage treatment (*n*=8) did not show survival benefit compared with salvage chemotherapy (*n*=12) for relapsed or refractory patients (*P=*0.675; Figure 3b). In this comparison, patients with early/hypoplastic death during induction therapy were excluded.

### Subgroup analysis for biologic prognostic factors of MK-AML

**Figure 4:**
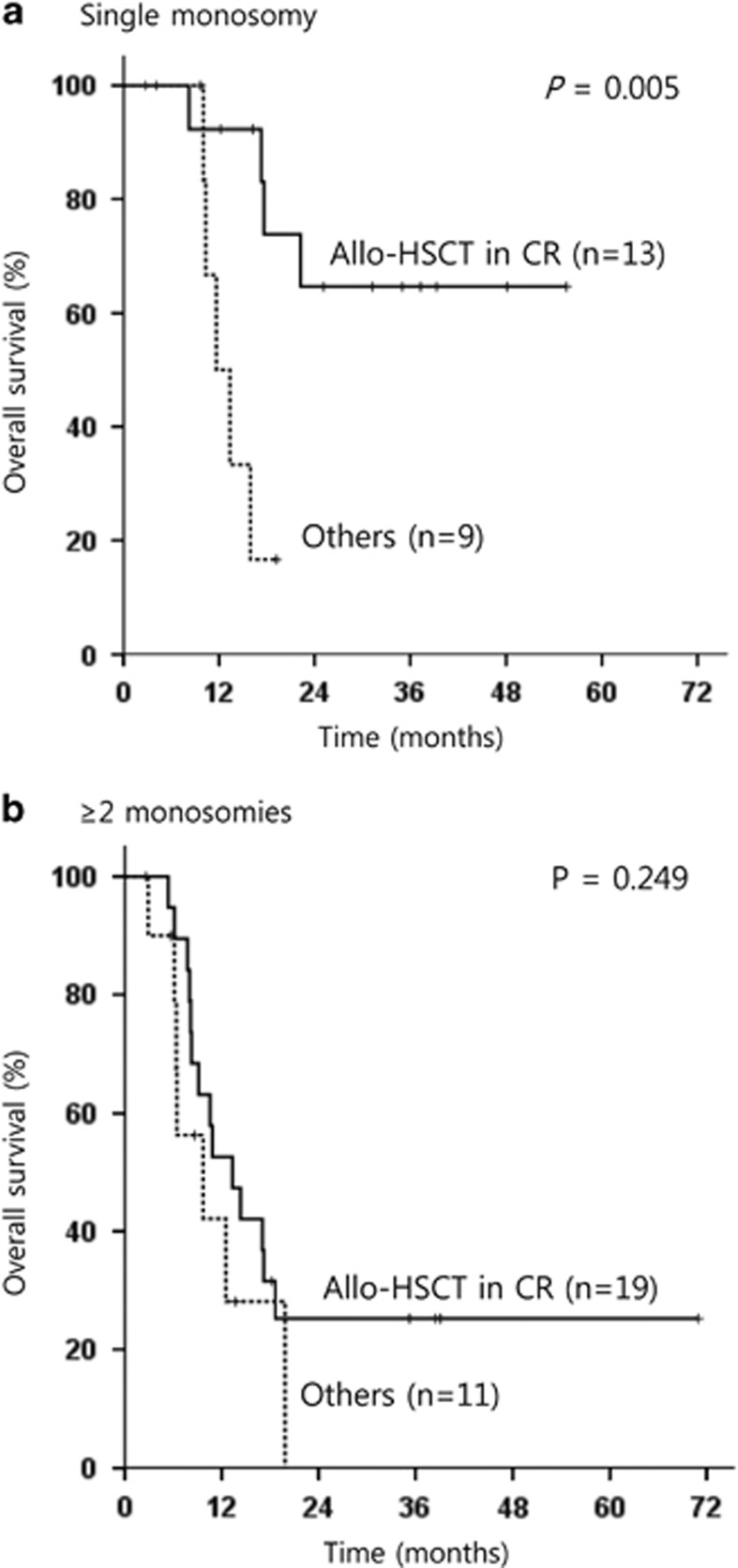
Kaplan–Meier estimate for OS according to the receipt of allo-HSCT in CR for consolidation in patients who achieved CR after induction therapy and (**a**) have a single monosomy or (**b**) have ⩾2 monosomies.

**Figure 5:**
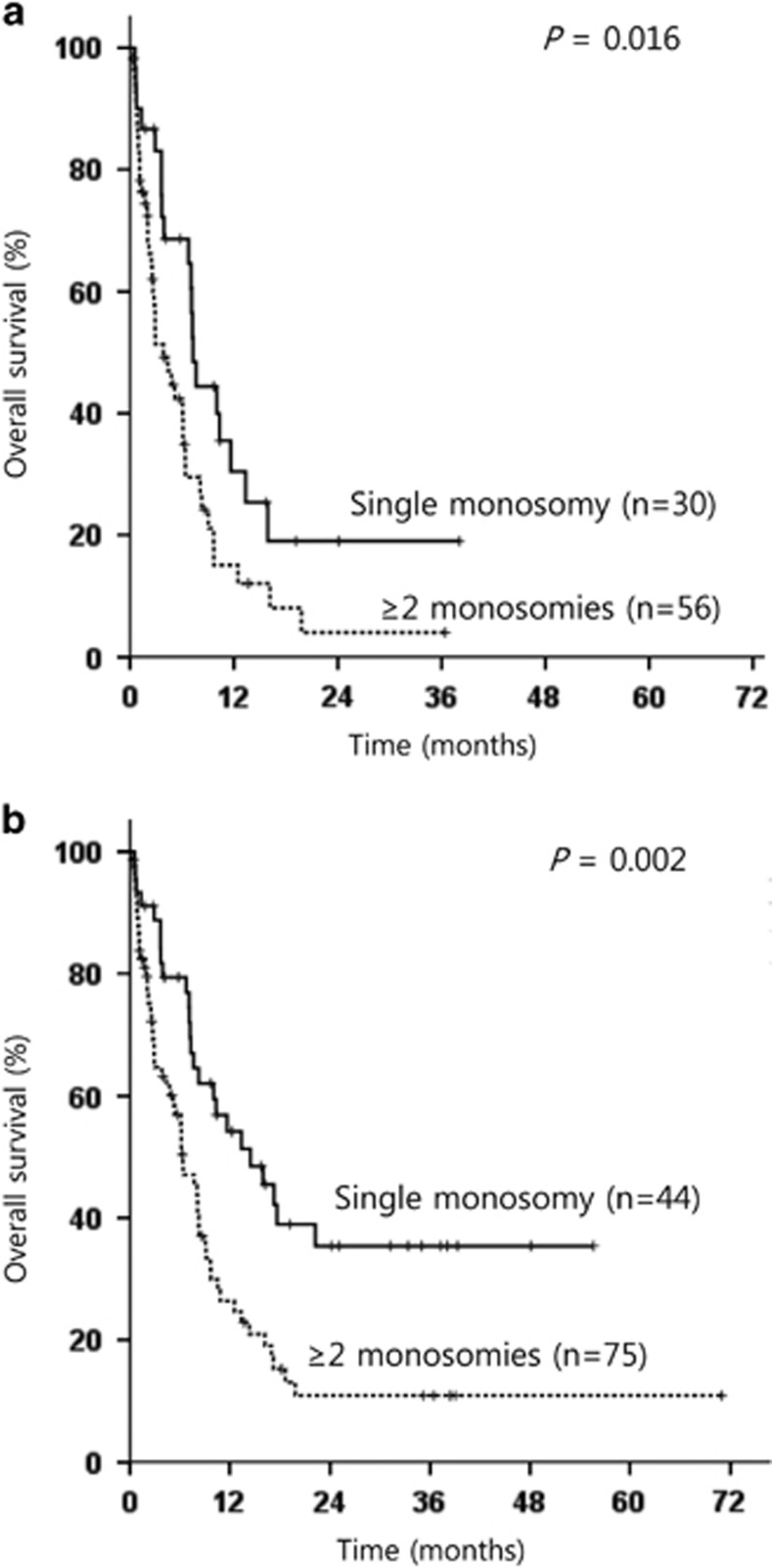
Kaplan–Meier estimate for OS according to the number of monosomies (**a**) in 86 patients, except those who received allo-HSCT in CR and (**b**) of 119 MK-AML patients.

Because allo-HSCT as postremission therapy has an important prognostic power for AML patients, we further performed subgroup analysis according to the type of postremission therapy. One patient who underwent transplantation in CR1 status after failing two cycles of induction therapy followed by salvage chemotherapy was excluded for analysis. Especially for 32 patients who received allo-HSCT in CR, univariate and multivariate analyses were performed for OS using Cox regression tests (Supplementary Table 4). The independent prognostic factor for a better OS for those was single monosomy subtype (HR: 0.273; 95% CI: 0.087–0.863; *P*=0.027). The beneficial impact of allo-HSCT in CR was not equally distributed in patients with single monosomy or ⩾2 monosomies. The 3-year OS after allo-HSCT in CR for patients with single monosomy was 64.6%, and allo-HSCT in CR improved OS significantly in patients with single monosomy (*P*=0.005; Figure 4a). However, in patients with ⩾2 monosomies, no beneficial impact of allo-HSCT could be demonstrated (*P*=0.249; Figure 4b). Next, another 86 patients who did not receive allo-HSCT in CR were analyzed to evaluate biological prognostic factors of MK-AML, excluding the therapeutic variable. Similar with the result of the analyses for the total 119 patients, multivariate analysis for these subgroup showed that the achievement of CR after induction therapy (*P*=0.002), single monosomy subtype (*P*=0.025), the presence of ⩾10% of cells with normal metaphase (*P*=0.019) and the absence of abn(17p) (*P*=0.027) correlated significantly with better OS rates (Supplementary Table 5). Patients with single monosomy showed superior OS compared with patients with ⩾2 monosomies, irrespective of the inclusion of patients who received allo-HSCT in CR (*P*=0.016 when these patients were excluded (Figure 5a) and *P*=0.002 (Figure 5b) when these patients were included).

## Discussion

In this study, we retrospectively analyzed MK-AML patients using a nationwide database from South Korean AML Registry to evaluate the predictive factors for better prognoses and to feature out clinical heterogeneity of patients according to the type of MK. MK-AML accounted for ~5.7% of Korean AML population, and was associated with lower CR rate after induction therapy and extremely poor outcomes, which is consistent with previous studies.^5, 7, 16^

Notwithstanding a dismal prognosis, multivariate analysis revealed that single monosomy, ⩾10% cells with normal metaphase, the absence of abn(17p) and achievement of CR after induction therapy were prognostic factors for better OS in Korean MK-AML patients. Single monosomy was also a prognostic factor for better OS in patients who received allo-HSCT in CR. The number of monosomies directly correlates with a poor prognosis in AML.^5, 6, 7^ To our knowledge, the significance of the prognostic value of a single monosomy in MK-AML has not been reported. In our study, patients in the ⩾2 monosomy group were older and had a higher incidence of abn(17p) and −5/del(5q), and in multivariate analysis of PFS, single monosomy remained as a significant factor for better PFS, whereas age, abn(17p) or −5/del(5q) had not a significant impact on PFS. The tumor suppressor gene *TP53*, located in the commonly deleted region, 17p13, is associated with a higher degree of genomic complexity and very poor prognosis.^17, 18, 19^ Several studies have reported that *TP53* mutations were associated with del(5q) or del(17p).^20, 21^ *TP53* alterations have been described in nearly 54–80% of MK-AML cases.^22^ A dysfunction in the *TP53* pathway contributes to an increase in chromosomal instability. The presence of abn(17p) is also an adverse risk factor in AML;^23, 24^ however, the significance of abn(17p) in MK-AML is not clear. Middeke *et al.*^10^ reported that MKs lose their poor prognostic value in patients who have undergone allo-HSCT when those with abn(17p) or −5/del(5q) are excluded. However, Breems *et al.*^25^ reported that MK retains its notoriously adverse prognostic value and does not depend on the inclusion of AML patients with abn(17p) and −5/del(5q). In our analysis, abn(17p), not −5/del(5q), had a significant adverse effect among MK-AML patients. The cohort of Middeke's study included elderly patients with a median age of 55 years with a range of 22–77 years, and the cohort of Breems's study included patients aged 15–60 years. The current study also included elderly patients with a wide age range. We found that patients with abn(17p) were significantly older than patients without abn(17p) in our cohort. Older MK-AML patients may be more affected by abn(17p) as the incidence is higher. Nevertheless, in multivariate analysis, abn(17p) still remained a significant impact factor for better OS, whereas age did not. The differences in prognosis between MK with single monosomy and ⩾2 monosomies could be biologically explained by the different incidence of the *TP53* mutation-associated chromosomal abnormalities in both groups. Further research has to be needed to determine which genomic alterations are mainly associated with the prognostic cytogenetic features demonstrated in this study.

In addition to a single monosomy and the absence of abn(17p), ⩾10% of cells with normal metaphase was also important prognostic factors. The presence of ⩾10% of cells with normal metaphase was a prognostic factor for OS and a significant contributor to achieving CR. A higher percent of normal cells in MK-AML has been reported to be associated with longer survival.^11^ How residual normal metaphases translate to longer survival is unclear. We demonstrated that having ⩾10% of cells with normal metaphase was associated with a higher rate of CR after induction therapy and longer OS. The fact that the achievement of CR is a critical factor for long-term survival in MK-AML may explain why having ⩾10% of cells with normal metaphase was associated with longer survival.

Allo-HSCT is currently the recommended consolidation treatment for poor-risk AML.^26, 27^ However, several studies have reported contrasting results regarding the benefit of allo-HSCT in patients achieving a CR after induction therapy,^7, 12, 13, 28^ and thus more research is necessary to define clearly the subgroups of MK-AML that would benefit from allo-HSCT. Our analysis demonstrated distinct differences in survival after allo-HSCT in CR depending on the karyotypic subtype of MK, single monosomy or ⩾2 monosomies. The beneficial role of allo-HSCT in CR was identified only in patients with single monosomy, and the 3-year OS of patients with single monosomy who received allo-HSCT in CR was 64.6% in this study. This survival outcome is comparable with the 3-year OS rate of patients without MK reported by Fang *et al.*^13^ Although it is hard to compare the results directly, MK-AML with single monosomy might need to be distinguished from the very poor-risk group.

In our study, because allo-HSCT in CR improved outcomes, we separately analyzed patients grouped by the receipt of allo-HSCT in CR to exclude the effect of the therapeutic factor in overcoming a poor prognosis. The independent prognostic factor in both groups was having a single monosomy. This result suggests that MK-AML with single monosomy may be biologically different from MK-AML with ⩾2 monosomies and the investigation of genetic differences is necessary.

This study has several limitations, including its retrospective design and the fact that the therapeutic strategies after induction therapy were chosen at the discretion of physicians and according to each institution's policy. However, therapeutic strategies in a single nation's medical system are relatively similar, and clinical variables that may affect clinical outcomes would be comparable among patient subgroups. Moreover, nationwide database, which was used for analysis in this study, was centrally collected by the Korean Society of Hematology to secure the objectivity.

In summary, although MK-AML was generally regarded as very poor-risk factor, patients with single monosomy, the absence of abn(17p) or ⩾10% of cells with normal metaphase experienced better prognosis than expected. Allo-HSCT had beneficial effect on prognosis when performed in CR status but not in relapsed/refractory status. However, Allo-HSCT in CR was associated with superior survival rates only in patients with a single monosomy. Interestingly, for those who did not receive allo-HSCT as postremission therapy, single monosomy was also an important favorable prognostic factor. Allo-HSCT in CR should be strongly recommended to MK-AML patients with a single monosomy, and for those with ⩾2 monosomies, more prudent treatment regimens are required. MK-AML with single monosomy might be biologically different from MK-AML with ⩾2 monosomies, and the biological mechanism by which these cytogenetic features influence patient prognosis should be further investigated.

## Figures and Tables

**Table 1:** Patient characteristics

	*Total patients*, n*=119*	*Single monosomy*, n*=44*	⩾*2 monosomies*, n*=75*	P*-value*
Median age (years) (range)	56 (17–82)	45.5 (17–78)	60 (23–82)	0.001
< 60, *n* (%)	69 (58.0)	34 (77.3)	35 (46.7)	
⩾ 60, *n* (%)	50 (42.0)	10 (22.7)	40 (53.3)	
				
*Sex*				
Male/female	83/36	26/18	57/18	0.064
				
*Type of AML*				
*De novo*, *n* (%)	100 (84.0)	38 (86.4)	62 (82.7)	0.595
Secondary, *n* (%)	19 (16.0)	6 (13.3)	13 (17.3)	
WBC ( × 10^9^/l) median (range) (*n*=118)	5.83 (0.54–316.2)	10.41 (0.54–85.15)	3.68 (0.96–316.2)	0.045
Hemoglobin (g/dl), median (range) (*n*=117)	8.2 (3.9–14.9)	8.5 (4.6–13.7)	8.1 (3.9–14.9)	0.133
Platelet count ( × 10^9^/l), median (range) (*n*=117)	55 (5–570)	70 (5–288)	52 (7–570)	0.033
PB blast, median % (range) (*n*=103)	29 (0–100)	35.5 (0–94)	25 (0–100)	0.149
BM blast, median % (range) (*n*=111)	52 (7.3–100)	61.3 (20–100)	44 (7.3–100)	0.189
				
*Cytogenetic abnormalities*				
Complex (⩾3 clonal abnormalities), *n* (%)	106 (89.1)	32 (72.7)	74 (98.7)	<0.001
Complex (⩾4 clonal abnormalities), *n* (%)	90 (75.6)	22 (50.0)	68 (90.7)	<0.001
Inv(3)/t(3;3), *n* (%)	8 (6.7)	6 (13.6)	2 (2.7)	0.050
Abn11q23, *n* (%)	1 (0.8)	1 (2.3)	0 (0)	0.370
Abn(17p), *n* (%)	21 (17.6)	2 (4.5)	19 (25.3)	0.004
t(6;9), *n* (%)	4 (3.4)	2 (4.5)	4 (2.7)	0.626
−5/del(5q), *n* (%)	49 (41.2)	7 (15.9)	42 (56.0)	<0.001
−7/del(7q), *n* (%)	56 (47.1)	19 (43.2)	37 (49.3)	0.516

Abbreviations: AML, acute myeloid leukemia; BM, bone marrow; PB, peripheral blood; WBC, white blood cell count; y, years.

**Table 2:** Multivariate analysis of prognostic factors for OS and EFS in 119 patients with MK-AML

	*Overall survival*		*Event-free survival*	
	*HR (95% CI)*	P-*value*	*HR (95% CI)*	P*-value*
Age (<60 years)	0.868 (0.540–1.397)	0.561	0.940 (0.609–1.449)	0.778
Single monosomy	0.460 (0.274–0.772)	0.003	0.514 (0.294–0.897)	0.019
Complex (⩾4 clonal abnormalities)	—		0.879 (0.481–1.607)	0.676
⩾10% Cells with normal metaphase	0.511 (0.311–0.841)	0.008	0.692 (0.447–1.071)	0.098
*De novo* AML	—		0.525 (0.297–0.930)	0.027
Absence of abn(17p)	0.532 (0.311–0.911)	0.022	0.708 (0.411–1.219)	0.213
Achievement of CR after induction Tx	0.238 (0.143–0.396)	<0.001	0.264 (0.168–0.416)	<0.001

Abbreviations: AML, acute myeloid leukemia; CI, confidence interval; CR, complete remission; EFS, event-free survival; HR, hazard ratio; MK, monosomal karyotype; OS, overall survival; Tx, therapy.

**Table 3:** Multivariate analysis of clinical outcome in 52 patients who achieved CR after induction therapy

	*Overall survival*		*Relapse-free survival*	
	*HR (95% CI)*	P-*value*	*HR (95% CI)*	P-*value*
Single monosomy	0.314 (0.135–0.732)	0.007	0.237 (0.092–0.615)	0.003
WBC <50 × 10^9^/l	—	—	0.177 (0.050–0.628)	0.007
*De novo* AML	—	—	0.098 (0.024–0.404)	0.001
Absence of abn(17p)	—	—	0.435 (0.164–1.152)	0.179
Allo-HSCT in CR	0.268 (0.090–0.798)	0.018	0.249 (0.099–0.626)	0.003

Abbreviations: Allo-HSCT, allogeneic hematopoietic stem cell transplantation; AML, acute myeloid leukemia; CI, confidence interval; CR, complete remission; HR, hazard ratio; WBC, white blood cell count.
