# Supplementary material for: Single monosomy as a relatively better survival factor in acute myeloid leukemia patients with monosomal karyotype
Source: Blood Cancer J. 2015 Oct 16;5(10):e358–. doi: 10.1038/bcj.2015.84 (PMC4635193; doi:10.1038/bcj.2015.84)
Supplement: Supplementary Tables [file bcj201584x1.pdf]

Table S1. CR rate after induction therapy and contributing factors

	n (%)	Univariate analysis		Multivariate analysis	
		OR (95%CI)	*P* value	OR (95%CI)	*P* value
All patients*	52 (46.0)				
Age, as a numerical variable (1 year older)		0.964 (0.939-0.990)	0.008	0.968 (0.942-0.996)	0.023
Age			0.111		
< 60	35 (52.2)	1.866 (0.867-4.017)		—	
≥ 60	17 (37.0)				
Number of monosomy			0.758		
1	22 (52.4)	1.100 (0.309-1.432)		—	
≥ 2	30 (42.3)				
Cells with normal metaphase			0.002		0.005
≥ 10%	27 (51.9)	3.626 (1.617-8.130)		3.263 (1.424-7.479)	
< 10%	25 (34.7)				
WBC, x10^9/L			0.743		
≥ 50	6 (50.0)	1.222 (0.369-4.051)		—	
< 50	45 (45.0)				
PB blast, %			0.511		
≤ 29 (median value)	21 (43.8)	1.310 (0.586-2.929)		—	
> 29	19 (37.3)				
BM blast, %			0.786		
> 52 (median value)	25 (48.1)	1.111 (0.520-2.376)		—	
≤ 52	25 (45.5)				
Type of AML			0.066		
De novo	47 (50.0)	2.800 (0.934-8.397)			
Secondary	5 (26.3)			—	
Complex (≥3 clonal abnormalities)			0.563		
Yes	48 (47.5)	1.419 (0.434-4.638)		—	
No	5 (38.5)				
Complex (≥4 clonal abnormalities)			0.626		
No	14 (50.0)	1.237 (0.526-23.909)		—	
Yes	38 (44.7)				
Abn(17p)			0.119		
No	46 (49.5)	2.284 (0.808-6.456)		—	
Yes	6 (30.0)				
-5/del(5q)			0.654		
No	32 (47.8)	1.189 (0.559-2.528)		—	
Yes	20 (43.5)				
-7/del(7q)			0.105		
No	31 (53.4)	1.859 (0.878-3.934)		—	
Yes	21 (38.2)				

CR: complete remission; WBC: white blood cell count; PB: peripheral blood; BM: bone marrow; AML: acute myeloid leukemia; OR: odds ratio; CI: confidence interval

* The total number of patients was 113 excluding 6 who were not available.

Table S2. Contributing factors for early/hypoplastic death

	Early/hypoplastic death, n (%)	*P* value
All patients	12 (10.6)	
Age		0.066
< 60	4 (6.0)	
≥ 60	8 (17.4)	
Number of monosomy		0.530
1	3 (7.1)	
≥ 2	9 (12.7)	
Cells with normal metaphase		0.033
≥ 10%	1 (2.4)	
< 10%	11 (15.3)	
WBC, x10^9/L		0.357
≥ 50	0 (0)	
< 50	12 (12.0)	
PB blast, %		0.831
≤ 29 (median value)	5 (10.4)	
> 29	6 (11.8)	
BM blast, %		0.926
≤ 52 (median value)	5 (9.1)	
> 52	5 (9.6)	
Type of AML		0.113
De novo	8 (8.4)	
Secondary	4 (21.1)	
Complex (≥3 clonal abnormalities)		0.626
Yes	10 (9.9)	
No	2 (15.4)	
Complex (≥4 clonal abnormalities)		0.727
Yes	10 (11.8)	
No	2 (7.1)	
Abn17p		0.037
No	7 (7.5)	
Yes	5 (25.0)	
-5/del(5q)		0.759
No	8 (11.9)	
Yes	4 (8.7)	
-7/del(7q)		0.608
No	7 (12.1)	
Yes	5 (9.1)	

WBC: white blood cell count; PB: peripheral blood; BM: bone marrow; AML: acute myeloid leukemia

Table S3. Univariate analysis of prognostic factors for OS and EFS in 119 patients with MK-AML

	**Overall survival**			**Event-free survival**		
	3y-OS (%)	HR (95%CI)	*P* value	3y-EFS (%)	HR (95%CI)	*P* value
All patients	19.6			7.3		
Median, months (95%CI)	8.1 (6.5-9.8)			4.6 (3.1-6.1)		
Age			0.022			0.052
< 60	24.1	0.596 (0.383-0.928)		9.9	0.674 (0.452-1.004)	
≥ 60	11.7			3.0		
Number of monosomy			0.002			0.002
1	33.5	0.474 (0.294-0.763)		16.4	0.510 (0.333-0.783)	
≥ 2	10.9			1.7		
Cells with normal metaphase			0.003			0.021
≥ 10%	30.8	0.489 (0.301-0.787)		7.6	0.619 (0.411-0.931)	
< 10%	12.6			6.3		
Type of AML			0.087			0.002
De novo	32.1	0.603 (0.338-1.075)		8.0	0.428 (0.249-0.737)	
Secondary	17.2			0		
WBC, $x10^9$/L			0.465			0.203
≥ 50	32.1	0.749 (0.345-1.627)		0		
< 50	17.2			8.1	0.648 (0.333-1.263)	
PB blast, %			0.316			0.828
≤ 29 (median value)	25.2	0.786 (0.490-1.259)		6.6	0.955 (0.629-1.449)	
> 29	12.8			6.0		
BM blast, %			0.448			0.064
> 52 (median value)	21.1	0.840 (0.534-1.319)		13.0	0.675 (0.445-1.023)	
≤ 52	15.6			2.5		
Complex (≥3 clonal abnormalities)			0.235			0.570
No	28.2	0.625 (0.288-1.357)		0	0.834 (0.445-1.561)	
Yes	18.5			7.8		
Complex (≥4 clonal abnormalities)			0.054			0.026
No	28.1	0.583 (0.337-1.009)		9.5	0.582 (0.362-0.937)	
Yes	17.0			6.6		
Abn(17p)			0.001			0.004
No	23.0	0.417 (0.248-0.701)		8.6	0.479 (0.290-0.793)	
Yes	5.1			0		
-5/del(5q)			0.139			0.152
No	23.6	0.716 (0.460-1.114)		8.7	0.749 (0.504-1.112)	
Yes	13.4			5.6		
-7/del(7q)			0.501			0.153
No	23.7	0.861 (0.556-1.332)		12.1	0.752 (0.508-1.112)	
Yes	15.2			2.3		
Achievement of CR after induction Tx*			<0.001			<0.001
Yes (n=52)	31.7	0.244 (0.151-0.395)		7.8	0.249 (0.164-0.380)	
No (n=61)	10.6			0		

OS: overall survival; EFS: event-free survival; MK: monosomal karyotype; AML: acute myeloid leukemia; HR: hazard ratio; CI: confidence interval; CR: complete remission; Tx: therapy

* Includes 113 patients, except those who were not available.

Table S4. Univariate and multivariate analyses of OS in 32 patients who achieved CR after induction therapy and received allo-HSCT in CR

		Univariate analysis		Multivariate analysis	
	3y-OS* (%)	HR (95%CI)	*P* value	HR (95%CI)	*P* value
All patietns	39.9				
Age			0.103		0.366
< 60	43.5	0.324 (0.083-1.256)		0.529 (0.133-2.107)	
≥ 60	25.0				
Number of monosomy			0.015	0.273 (0.087-0.863)	0.027
1	64.6	0.025 (0.082-0.768)			
≥ 2	23.4				
Cells with normal metaphase			0.832	—	—
< 10%	42.9	0.904 (0.355-2.303)			
≥ 10%	37.5				
Donor			0.832	—	—
Matched related	40.0	1.290 (0.483-3.448)			
Matched unrelated	38.5	0.938 (.0368-2.387)			
Haploidentical	37.5	0.709 (0.161-3.116)			
Conditioning			0.536	—	—
MA	44.6	0.746 (0.295-1.889)			
RIC	34.3				
Status at transplantation			0.732	—	—
CR2	66.7	0.703 (0.093-5.294)			
CR1	38.4				
Acute GVHD			0.878	—	—
Yes	39.7	0.930 (0.366-2.364)			
No	40.4				
Chronic GVHD			0.282	—	—
Yes	62.5	0.488 (0.132-1.806)			
No	42.1				

Allo-HSCT: allogeneic hematopoietic stem cell transplantation; MA: myeloablative; RIC: reduced intensity conditioning; CR: complete remission; OS: overall survival; HR: hazard ratio; CI: confidence interval; GVHD: graft versus host disease

[*] OS from the date of transplantation.

Table S5. Multivariate analysis of OS in 86 patients, except those who received allo-HSCT in CR

	**Overall survival**		**Event-free survival**	
	HR (95%CI)	*P* value	HR (95%CI)	*P* value
Age (< 60 years)	1.101 (0.626-1.935)	0.739	1.327 (0.793-2.218)	0.281
Single monosomy	0.480 (0.261-0.880)	0.018	0.541 (0.316-0.927)	0.025
Complex (≥ 4 clonal abnormalities)	—	—	—	—
≥ 10% Cells with normal metaphase	0.455 (0.243-0.852)	0.014	0.542 (0.306-0.961)	0.036
De novo AML	—	—	—	—
Absence of Abn(17p)	0.534 (0.295-0.968)	0.039	—	—
Achievement of CR after induction Tx	0.333 (0.167-0.665)	0.002	0.411 (0.223-0.755)	0.004

Allo-HSCT: allogeneic hematopoietic stem cell transplantation; CR: complete remission; OS: overall survival; HR: hazard ratio; CI: confidence interval; Tx: therapy
